# Interpretable generative deep learning: an illustration with single cell gene expression data

**DOI:** 10.1007/s00439-021-02417-6

**Published:** 2022-01-06

**Authors:** Martin Treppner, Harald Binder, Moritz Hess

**Affiliations:** 1 Institute of Medical Biometry and Statistics, Faculty of Medicine and Medical Center, University of Freiburg, Stefan-Meier-Str. 26, Freiburg, 79104 Germany; 2 Freiburg Center for Data Analysis and Modeling, University of Freiburg, Freiburg, 79104 Germany

**Keywords:** Explainable AI, Deep learning, Generative model, Dimension reduction

## Abstract

Deep generative models can learn the underlying structure, such as pathways or gene programs, from omics data. We provide an introduction as well as an overview of such techniques, specifically illustrating their use with single-cell gene expression data. For example, the low dimensional latent representations offered by various approaches, such as variational auto-encoders, are useful to get a better understanding of the relations between observed gene expressions and experimental factors or phenotypes. Furthermore, by providing a generative model for the latent and observed variables, deep generative models can generate synthetic observations, which allow us to assess the uncertainty in the learned representations. While deep generative models are useful to learn the structure of high-dimensional omics data by efficiently capturing non-linear dependencies between genes, they are sometimes difficult to interpret due to their neural network building blocks. More precisely, to understand the relationship between learned latent variables and observed variables, e.g., gene transcript abundances and external phenotypes, is difficult. Therefore, we also illustrate current approaches that allow us to infer the relationship between learned latent variables and observed variables as well as external phenotypes. Thereby, we render deep learning approaches more interpretable. In an application with single-cell gene expression data, we demonstrate the utility of the discussed methods.

## Introduction

Omics data allow to characterize individuals genetically and phenotypically in high resolution. Since almost two decades, high-resolution genetic data is now employed in genome-wide association studies (Bush and Moore 2012) to examine associations of single nucleotide polymorphisms (SNPs) with phenotypes. In recent studies, investigations have expanded to include high-dimensional phenotypes, e.g., gene expression in expression quantitative loci (eQTL) studies (Gilad et al. 2008) or metabolites (Suhre et al. 2011). These high-resolution data offer a fundamentally better understanding of the heritability of traits or causal factors for disease susceptibility. However, an interpretation of the results derived from this sheer mass of data is challenging. The lack of established methods for the interpretable analysis of such data is one reason. Routinely, pairwise associations between individual SNPs and the high dimensional phenotypes such as gene expression are inferred. This confronts the researchers with potentially millions of statistics that ignore the interdependency of the genotypical and phenotypical variables.

However, it is reasonable to assume a high amount of redundancy in the genotype and phenotype data. Therefore, a small number of variables can capture the essential variation in the data. If we assume a linear relation between the observed variables and the variables of a dimension-reduced latent representation, principal component analysis (PCA) can be used for dimension reduction. However, if non-linear relations should be allowed, deep generative models (DGMs), such as variational autoencoders (VAEs) (Kingma and Welling 2013), deep Boltzmann machines (DBMs) (Salakhutdinov and Hinton 2009), or generative adversarial networks (GANs) (Goodfellow et al. 2014), provide a potential solution. In the following, we provide an overview of such techniques. As deep learning approaches have been criticised for being hard to interpret, we also introduce approaches for interpretability. In an exemplary application for single-cell gene expression data, we want to illustrate how such techniques can be useful to more generally stimulate use for omics data.

A probabilistic, parameterized model allows for better generalizations and better flexibility. In fact, deep generative models have been successfully applied in a number of scenarios. For example, Xu et al. (2021) integrated gene expression profiles from single cells of different experimental origins. Here, DGMs were employed to give a continuous indicator of how likely an inferred label was, given the omics information. Similarly, Kim et al. (2020) proposed to employ DGMs for improved predictions based on omics data by integrating different data sources.

Deep generative models can also be employed as an extension to shallow latent variable models, which have been proposed to account for gene expression heterogeneity related to nuisance factors (Yang et al. 2013; Stegle et al. 2010). Specifically, in Lopez et al. (2018), DGMs were employed to extract sources of technical variation from the data.

In addition to gene expression data, DGMs have also been successfully applied to high-dimensional SNP data. Montaez et al. (2018) employed deep learning approaches for performing the joint analysis of multiple SNP loci, thereby allowing the researcher to study multigenic diseases. Riesselman et al. (2018) used generative models for predicting the effects of nucleotide changes on the DNA level. A comprehensive overview of deep learning for SNP data can be found in Azodi et al. (2020).

In the following, we first briefly describe building blocks such as artificial neural networks, autoencoders, and corresponding model-fitting approaches. Secondly, we provide an overview of popular deep generative approaches. We then discuss adaptations to omics data, specifically single-cell RNA-sequencing (scRNA-seq) data. Afterwards, we discuss current approaches for rendering DGMs interpretable. This involves methods for identifying components of the latent space that relate to specific external phenotype data, as well as methods for inferring the observed variables that relate to the latent variables. We also provide exemplary applications for two interpretable DGMs. These applications can be recapitulated in supplementary Jupyter notebooks.

**Fig. 1:**
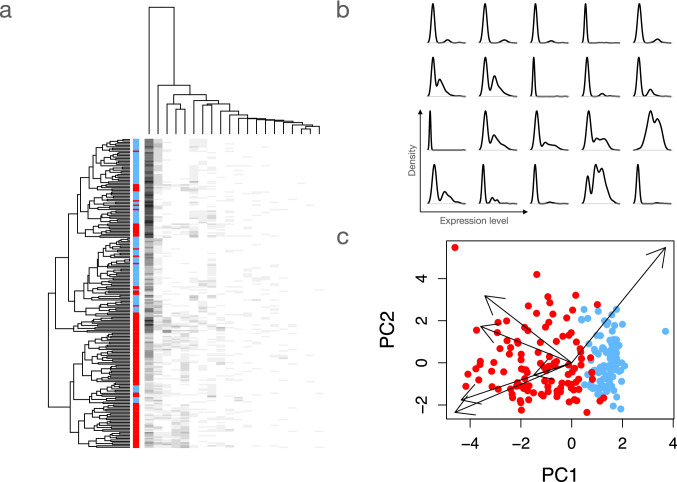
Dimension reduction and structure of omics data. Dimension reduction aims at extracting a low-dimensional representation from high-dimensional data. **a** Observations of single-cell RNA-sequencing (scRNA-seq) count data. Twenty variables (columns) are studied in about 200 observations (rows). Hierarchical clustering of log-transformed counts reveals a crude discrimination between two groups of cells (blue and red labels). Still, an interpretation of how genes contribute to the clusters seems difficult. **b** In scRNA-Seq data, genes are often bimodally expressed. Expression is very low or absent in a large number of cells, while higher levels corresponding to an active state are observed in a smaller proportion. Density estimates are shown for the 20 genes presented in (**a**). **c** Principal components analysis (PCA) is usually applied for extracting an interpretable representation from omics data. In the graphic depicted above, the two PCs which capture the highest proportion of variation are shown. Each dot corresponds to the values of PC1 and PC2 for a single cell. Since PCA is, to a large extent, equivalent to a linear factor model, the contribution of the observed variables (genes) to the PCs can be straightforward extracted (arrows). Shown are six exemplary genes. Each arrow corresponds to a gene and the direction of the arrow indicates how the genes are related to the PCs. Specifically, an arrow indicates how a single cell would move in the two-dimensional PC space if the expression of the corresponding gene would change. The length of the arrows indicates how sensitive the movement is with respect to the change in gene expression levels

## Building blocks for dimension reduction by deep neural networks

In all dimension reduction techniques, from principal components analysis (PCA) to deep neural networks, we assume that the measured variables, e.g., the gene expression profiles of cells of a group of patients, do not optimally capture the underlying structure in the data because much of the underlying information is hidden by noise and redundancy. For high dimensional omics data, this means that the similarity of observations, e.g., patients, can be sufficiently well described by a small number of latent variables learned from a dimension reduction approach. PCA, for instance, transforms the original coordinate system defined by the observed variables into a new one. In the case of multivariate normally distributed data, the new axes, namely, the principal components (PCs), are all orthogonal to each other and are sorted according to the variance they explain. Using only the PCs that explain the highest amount of variance then allows for studying the structure in the data in a low dimensional space (Fig. 1c). Similar approaches such as nonnegative matrix factorization (NMF) (Lee et al. 1999) use different objective functions. Nevertheless, they have in common with PCA that they only capture linear relationships. Non-linear variants of PCA have been proposed but usually fail in real-world applications (Van Der et al. 2009).

### Artificial neural networks

**Fig. 2:**
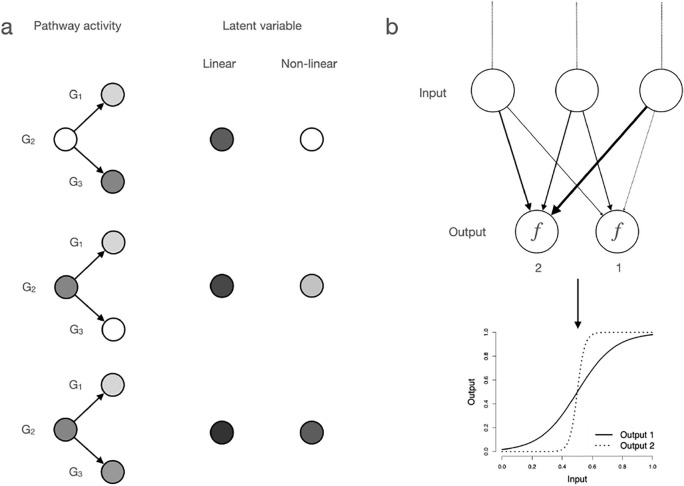
Capturing non-linearity with deep neural networks. **a** Example of a of non-linear relationship of gene products. Three genes are involved in a regulatory circuit. $G_1$ and $G_3$ are only functional if $G_2$ is expressed. In this graphic, two examples for latent variables which are / are not able to capture the non-linear dependency are shown. Color intensity of genes indicates transcript abundance as e.g. measured by scRNA-seq. Color intensity of latent variables represents dimensionless values. Arrows indicate the post-transcriptional dependency. **b** Deep neural networks can capture non-linearities by non-linear activation functions. In this example, the output of two neurons conditional on their input is shown. The weights (arrows) connecting neuron 2 with its input neurons are larger than those for neuron 1. This means that neuron 2 responds much more sensitively to activations of input neurons. Specifically, neuron 2 closely resembles a step function that is active if some level in the input neurons is reached. In contrast, neuron 1 responds more linearly to its input. In a simplified example, neuron 2 might be active when some genes in a pathway are expressed beyond a given expression level. This gene expression pattern might be frequently observed in the training data, which led to the learning of the respective high weights. Note that the input of the two neurons is shown normalized

**Fig. 3:**
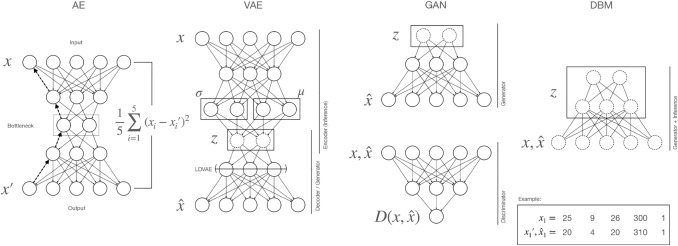
Autoencoder and deep generative approaches. An Autoencoder (**AE**) is trained in an unsupervised manner to minimize the error between a reconstruction $x^\prime$ and the input data *x* used for training. Usually, the mean squared error is employed (see formula). The loss is minimized by computing the gradient for the network parameters, e.g., the weights, indicated here as arrows. In this example, all weights are equal, indicated by the identical thickness of lines. The gradient indicates to what extent the loss is dependent on the respective parameter. The loss computed for the terminal layer ($x^\prime$) is propagated back through the network (exemplary indicated for some neurons by dashed arrows) to calculate the dependence on neurons in the intermediate (hidden) layers. DGM approaches have in common that they employ a number of random variables (indicated by dashed lines) *z* (and *x* in case of the DBM) to generate synthetic data (${\hat{x}}$), which should mimic the training data (*x*). In the **VAE** a distribution is learned over the variables in the bottleneck layer (*z*). During training, the distribution is regularized to follow a multivariate normal distribution with diagonal covariance, which is parameterized by a mean and standard deviation represented by the neurons $\mu$ and $\sigma$. These are also termed the variational parameters. A neural network, also termed encoder, parametrizes the distribution of *z*. The latent representation (*z*) is transformed to the space of the observed variables with the generator network (decoder). Like the VAE, the **GAN** also has a generator network, but here, no encoder is available that learns the latent representation. Instead, a supervised encoder network (discriminator) computes a loss which indicates if the generated examples ${\hat{x}}$ can be discriminated from real data (*x*). A gradient of the generator parameters with respect to the loss is then used to update the weights of the generator to generate examples that the discriminator cannot discriminate from real data. The parameters of the discriminator are updated as well to compete with the improved generator. **DBMs** deviate from GANs and VAEs in their general design. In contrast to those mentioned above, they are undirected models and consist only of random variables. $x_1$,$\hat{x_1}$, and $x^{\prime }_{1}$ are a training example, a synthetic sample or a reconstruction respectively

We consider an example for a non-linear relationship, e.g., of gene expression levels (Fig. 2a). Specifically, expression data for three genes ($G_1$ to $G_3$) which are involved in a regulatory circuit are available. We now want to model the pathway activity which is not directly observed and is to be estimated from the gene expression data. The biochemical function of $G_1$ and $G_3$ depends on $G_2$. For example, $G_2$ might be a co-factor and $G_1$ and $G_3$ might be transcription factors. If we would model the pathway activity with a linear technique such as PCA, and want to model the pathway activity with a single variable, we could only infer the linear combination of the gene expression levels of $G_1$ to $G_3$. In this example, we are unable to discriminate between an activated and non-activated regulatory component, since the values of the latent variables are more or less equal. A dimension reduction technique which can model the above-described non-linear pattern, however, could infer a single latent variable which indicates if $G_2$ is expressed. The value observed for the latent variable could then represent the expression level of $G_1$ and $G_3$ under the condition that $G_2$ is expressed. Deep neural networks are able to learn such a pattern since they are able to approximate any complex function through an ensemble of latent variables, namely, neurons, which are arranged in layers (Hornik et al. 1989) (Figs. 2b and 3). Connections between neurons of the same layer are usually absent. The underlying principle of learning non-linear relationships with neural networks is that individual neurons are activated when a weighted sum of the states of connected neurons reaches a given threshold. The strength with which two neurons are connected to each other is termed ”weight” and is a dimensionless value. To make training feasible, no hard threshold is explicitly employed. Instead, a non-linear activation function is used, which transforms the weighted sum into a non-linear response. In case of the sigmoid function, which was frequently employed in the early implementations of neural networks, the input is transformed into a range of 0–1 (Fig. 2b). The weighted sum then defines the sensitivity with which the output of a neuron responds to the input. The higher the input, the more the output resembles a binary step function (compare outputs 1 and 2 in Fig. 2b). Today, rectified linear units (ReLU) are frequently employed. In contrast to the sigmoid function, their output is not bounded, while otherwise the same principles apply.

### Autoencoder (AE)

An AE is a neural network that learns a low dimensional representation from the training data in an unsupervised way (for a popular application, see Hinton and Salakhutdinov (2006)). The AE contains a number of layers of latent variables (neurons) (Fig. 3). The dimension reduction is achieved by reducing the number of latent variables in an intermediate layer. This layer is also frequently referred to as the bottleneck layer (Fig. 3). The multiple layers of latent variables with non-linear activation functions in the AE allow to learn interactions of the input variables. For example, one neuron in the bottleneck layer could represent the non-linear pattern shown in Fig. 2a. An AE is trained in an unsupervised manner. Specifically, training examples, i.e., gene expressions of a single cell, are fed into the input layer of the AE. By multiplication with the weights, which indicate the strength with which two neurons are connected, the values for all latent variables and the variables in the output layer are subsequently calculated (Fig. 3). The objective function of an AE is then to minimize the reconstruction error for the training data. This is achieved by comparing the reconstruction of the input $x^\prime$ with the original input *x* (Fig. 3). Since the number of parameters in an AE is usually much larger than the number of observations, the AE is prone to overfitting. To avoid overfitting, regularization can be employed with different approaches, one popular of them being the dropout technique (Srivastava et al. 2014). During training, a random selection of neurons is ignored from the fitting process. This introduces noise during the fitting process, which makes the training more robust. The approach has some conceptual similarities with random forests (Breiman 2001) which avoid overfitting of partition trees by considering only a random selection of variables at each partition. A technique, similar to the dropout technique is employed in denoising AEs. In contrast to dropout, no latent neurons are removed during the fitting process, but input nodes are set to zero. This results in an AE, which learns to reconstruct potentially missing observations (Vincent et al. 2008).

### Fitting deep neural networks

Fitting a deep neural network to data is generally more complex compared to shallow approaches such as linear regression models. The reason is that not only the weights of observed variables have to be estimated, e.g., to predict the value of a response variable. In addition, we have to infer the states of the latent variables in the hidden layers. Let us consider the training of an AE as an example. Our goal is to minimize the reconstruction error, the loss, which is calculated from the input and output layers of the AE. In order to derive an AE whose reconstructions are similar to the input, resulting in a low loss, we have to get meaningful values for the weights that connect the neurons in the different layers. Specifically, we need information about how sensitive the reconstruction error is to a change in the weights that connect input, hidden as well as output neurons. This information is termed the gradient. The gradient is, due to time constraints, usually evaluated based on a small fraction of the training data, which is termed stochastic gradient descent (SGD) (explained in Bottou (2012)). SGD is an iterative procedure. Model parameters are optimized by feeding the training data in multiple iterations (epochs) to the model. Parameters are updated by small proportions of the gradient, while the magnitude of the proportion is also termed ”learning-rate”. To adjust the weights not only for the terminal layer but also for all layers in between, a technique called backpropagation of errors (Rumelhart et al. 1986) is employed. Here, the gradient, evaluated at the terminal layer, is decomposed to reflect the contribution of each neuron in the complete network. As the gradient is the derivative of the loss function with respect to the network parameters, this decomposition is achieved by the chain rule.

## Deep generative models

**Fig. 4:**
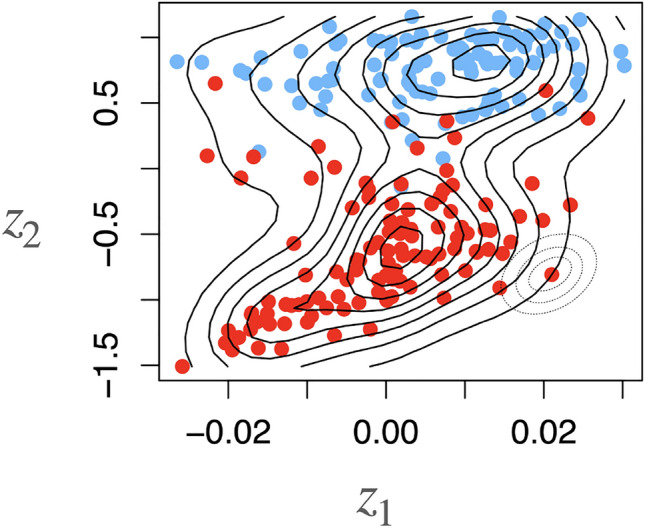
Dimension reduction with DGMs. Example for a two-dimensional latent representation of the data shown in Fig. 1 learned by a DGM. Notice the compared to PCA (Fig. 1) better linear separability of the observations. Contours shown in the figure indicate that a distribution over the latent variables has been learned. This implies, that we can get the uncertainty for observations in the latent space, also indicated by the concentric rings plotted around a red observation in the bottom right

Deep generative models (DGMs) introduce random latent and observed variables, which allow learning a distribution over both (Fig. 3). They explicitly or implicitly maximize a likelihood lower bound for the training data, which means that the likelihood of the training data can be maximized up to a certain amount. The likelihood indicates how well a data point, i.e., a patient instance or cell, is supported by the model. With regards to deep generative models, this could also be termed as: ”How likely is it that a given data point has been generated by the model”. This implies that a likelihood can be assigned to a single data point, e.g., as performed in Ding et al. (2018; Fig. 4).

Deep generative approaches have been successfully applied in many fields of gene expression related research. For an overview, see e.g., Lopez et al. (2020).

### Fitting deep generative models

Since deep generative models learn a distribution over the observed and latent variables, potentially very complex distributions have to be approximated. Consider, e.g., (Gaussian) mixture models that estimate the parameters of a mixture of (Gaussian) distributions. The probabilities with which observations are assigned to the components of the mixture can be seen as latent factors that have to be inferred. In the popular expectation-maximization (EM) algorithm (Dempster et al. 1977), values for the latent variables computed in a current iteration are plugged in for an iterative optimization. For deep generative approaches, this approach is not possible because the values of the latent variables can not exactly be computed. Instead, they have to be approximated. For approximation, there are two prominent approaches termed (Bayesian) variational inference (Blei et al. 2017) and Gibbs sampling (explained in Resnik and Hardisty (2010)). In variational inference, we assume that the distribution of the latent variables is related to a simple distribution (prior distribution). During training, we condition on the prior distribution to infer the posterior distribution of the latent variables. Specifically, we get an approximation for the distribution of the latent variables, given the training (here gene expression) data. Gibbs sampling is a Markov chain Monte Carlo (MCMC) technique. In DGMs, the objective is to infer the joint distribution of observed and latent variables. This is achieved by estimating a series of conditional expectations for latent and observed variables. Specifically, in each iteration, the states of all neurons are sequentially inferred by conditioning on the states of all other neurons. After performing the procedure for several iterations, we arrive at an estimate for the joint distribution of observed and latent variables. The absence of lateral connections in a neural network makes Gibbs sampling comparatively efficient. Nevertheless, this approach is still considerably slower than variational inference, especially when a large number of observed and latent variables are studied. The above presented approaches to approximate the joint distribution of observed and latent variables indicate a close relationship between the training of DGMs and generating synthetic samples.

### Variational autoencoder (VAE)

VAEs (Kingma and Welling 2013) are, given the architecture of the neural network, similar to the AE, but their training objective differs fundamentally. AEs aim to learn a reconstruction of the data, which generalizes well to new unseen data. As a by-product, a low dimensional representation can be learned by introducing a bottleneck layer of significantly lower dimensionality compared to the dimensionality of the original training data. In contrast, the objective of the VAE is to directly learn a posterior distribution over latent variables, which represent the latent structure of the observed data. As the VAE learns a distribution, we can draw samples from the distribution and thereby get information about uncertainty in the data. The distribution of the latent variables is inferred by variational inference. As a prior distribution, researchers often use a multivariate standard Gaussian distribution. In the VAE, there are two neural networks, the inference (encoder) and the generator (decoder) network (Fig. 3). The inference network is trained to learn the parameters of the distribution over the latent variables, while the generator network is trained to learn a transformation from the latent variables to the observed variables. The parameters of the inference and the generator network are updated by a loss which is composed of two factors. One factor ensures that the generated synthetic observations are similar to the training data by employing the reconstruction error (see Fig. 2a). The other factor assures that the distribution estimated for the latent variables is similar to the employed prior distribution. This is achieved by the Kullback–Leibler divergence (Kullback and Leibler 1951) which is used to measure the deviation from the prior distribution. As the learned distribution over the latent space *z* is regularized to mimic a Gaussian distribution with diagonal covariance, the latent variables in *z* should be more or less independent. The amount of regularization can be controlled by increasing the influence of the deviation from the prior distribution on the loss. This principle is employed in the $\beta$-VAE (Higgins et al. 2016) which has also been proposed as an approach for increasing the interpretability of VAEs. VAEs have been observed to learn the joint distribution even from small amounts of data with a low amount of variance (Nußberger et al. 2020).

### Generative adversarial network (GAN)

A generative adversarial network (GAN) (Goodfellow et al. 2014), similar to VAEs, employs feed-forward neural networks for learning a transformation between the observed and latent variables. Similar to the VAE, a generative component (generator) is present (Fig. 3). This neural network transforms samples from a simple distribution, such as a multivariate normal distribution with a diagonal covariance matrix, into a representation of the same dimensionality as the training data. However, the training objective differs fundamentally from the VAE. While in the VAE, there is an explicitly formulated objective for maximizing the likelihood of the training data, meaning that for data points, a probability given the model parameters can be stated, the maximization of the likelihood is implicitly achieved in the GAN. Specifically, the GAN possesses a discriminator network which can be seen as a supervised neural network. The objective of this network is to discriminate between real data points and synthetic data points sampled from the generator. During training, the discriminator and the generator compete against each other. The generator aims at generating synthetic observations which the discriminator cannot discriminate from real observations. The discriminator then assigns a score to each presented synthetic or real data point. When the generator is sufficiently accurate in generating synthetic data that the discriminator cannot discriminate from real data, a value of 0.5 is returned for both in case the score ranges from 0 to 1, which is the case for the classic GAN that builds on the Kullback-Leibler divergence. While classic GANs are difficult to train, using the Wasserstein metric has been proposed in the Wasserstein GAN (Arjovsky et al. 2017). Compared to DBMs and VAEs, GANs have been described to be difficult to adapt to omics data with a small sample size (Nußberger et al. 2020). Nevertheless, Xu et al. (2020) successfully employed GANs for imputing gene expression data.

### Deep Boltzmann machine (DBM)

The deep Boltzmann machine (Salakhutdinov and Hinton 2009) represents a different approach compared to VAE and GAN. Similar to the VAE and GAN, the training objective is to learn weights so that the samples drawn from the DBM have the same properties as found in the training data. Explicitly, the weights are optimized according to a gradient formed between states of the observed and latent variables calculated for the training data and a sample from the DBM. Samples from the DBM are retrieved by Gibbs sampling from the distribution formed over all observed and latent variables. Thereby, the likelihood for the presented data is increased while simultaneously reducing the likelihood for the randomly selected states. DBMs have been shown to work particularly well for binary omics data, such as haploid SNP data (Nußberger et al. 2020).

## Adaptation of DGMs to omics data

### Modeling count data

Among all omics-type measurements, scRNA-seq is the most frequently studied, e.g., in single-cell eQTL approaches (Majumdar et al. 2020). Typically, a scRNA-seq protocol involves the isolation of single cells and their messenger RNA (mRNA), reverse transcription, library preparation, and sequencing (Kim et al. 2015). The experimenters obtain counts of how many of the fragments have been mapped to a particular gene, resulting in a large cell by gene count matrix. The experimental steps and the resulting sampling process in scRNA-seq studies were commonly modeled using the Poisson distribution, which was shown to give a good description of the technical noise in scRNA-seq data (Grün et al. 2014; Kim et al. 2015; Wang et al. 2018). However, the assumptions accompanying the Poisson distribution often do not match real observations. Usually, our datasets contain heterogeneous cell populations or heterogeneity due to biological processes like cell cycle, which increase the dispersion (width) of the distribution (Kim et al. 2015). This can lead to excess variability, also referred to as overdispersion, which the Poisson distribution might not be able to model (Hilbe 2011). To account for this overdispersion, we have to add an additional layer of modeling. Hence, most researchers use the negative binomial distribution (also known as gamma-Poisson mixture), which captures technical and biological variability, to describe scRNA-seq data. Additionally, different experimental protocols might also lead to varying levels of zero counts, the modeling of which has been widely investigated (Hicks et al. 2018; Townes et al. 2019; Silverman et al. 2020; Svensson 2020). The presence of many zero counts and biological variability often leads to bimodal distributions in scRNA-seq data (Fig. 1b).

### Single cell variational inference (scVI)

A framework to adapt the classical VAE to the characteristics of count data resulting from scRNA-seq experiments was developed by Lopez et al. (2018). Just like the VAE, in scVI, the data is encoded into a lower-dimensional latent representation and reconstructed via a decoder network (see VAE in Fig. 3). Two neural networks are employed to estimate the parameters of the negative binomial distribution. Additionally, the model can estimate the parameters for a zero-inflation term, allowing for acknowledging a higher number of zeros than expected. Synthetic scRNA-seq data can be generated in scVI by the following process. In the latent space of scVI, samples are drawn from a multivariate normal distribution. The samples are then, together with batch information, where each cell is assigned membership to an experimental batch such as a well plate in which cells have been processed, fed into two decoder networks. One of the networks estimates the expected number of transcripts across all genes, and the other estimates whether a dropout event has occurred. As described above, scRNA-seq data can be modeled by a gamma-Poisson mixture. Hence, the expected number of transcripts for each gene is entered into a gamma distribution, and the corresponding inverse dispersion is estimated using variational inference. Thereby, the overdispersion, which can occur, e.g., through heterogeneous groups of cells, is taken into account. The resulting random variable is then multiplied by a library-size scaling factor and used as the rate parameter of a Poisson distribution. This mixture, resulting in a negative binomial distribution, is the output of the generative process in scVI (Lopez et al. 2018).

The learned lower-dimensional latent representation of scVI can give insights into the molecular phenotypes of cells through an unsupervised learning approach. These representations learned through the encoder can be used for downstream analyses to aid interpretability (Lopez et al. 2018). For example, representations can be visualized in two dimensions and then colored with the help of known factors, e.g., batch information, to get a first impression of whether this factor leads to increased variation in the data (see, e.g., Fig. 4). Additionally, by accounting for technical factors in the lower-dimensional latent representations, one can improve the interpretability of the models, as the biological signal can be extracted more clearly (Lopez et al. 2020).

Due to the probabilistic orientation of scVI, researchers can also use the decoder for statistical inference. For example, Lopez et al. (2018) show that the model can be used to test differential expression between the genes of two groups of cells while also accounting for potential technical effects. Boyeau et al. (2019) have extended scVI with a more complex Bayesian approach to test differential expression.

So far, the lower-dimensional latent representations learned by scVI have primarily been used to correct for batch effects, i.e., systematic differences between, e.g., well plates during the execution of an experiment, and to integrate biological data from different modalities. For example, Gupta et al. (2021) investigated single-nuclei RNA-sequencing (snRNA-seq) for its ability to identify cell types at different states of adipogenesis. They used scVI for removing technical batch effects which facilitated integrating snRNA-seq and scRNA-seq data for inferring joint patterns between both data types. As a result, they were able to detect shared cell populations across the different sequencing techniques which was impossible otherwise. Govek et al. (2021) used scVI to retrieve a lower-dimensional representation of a CITE-seq dataset of the murine spleen. This representation was helpful in further downstream analyses such as clustering and differential expression analysis. Similarly, Quinn et al. (2021) and Schupp et al. (2000) made use of scVIs ability to learn complex non-linear representations in cancer cells and lung epithelial cells, respectively.

Gayoso et al. (2021) provide a comprehensive and highly optimized package called scvi-tools with which scientists can perform end-to-end analyses. They also offer tutorials on using the corresponding methods and ensure integration with other common analysis methods such as Seurat (Stuart et al. 2019), and Scanpy (Wolf et al. 2018).

## Interpretable AI

As defined by Montavon et al. (2018), an interpretable representation is a representation that can somehow be understood by humans. For example, a two-dimensional representation retrieved by PCA yields an interpretable representation because we can visually inspect if samples with a given label are more or less similar to each other, given their scores on e.g., two plotted principal components (see Fig. 1). Compared to PCA, DGMs can more efficiently capture the essential structure, which reduces the number of latent variables to be studied and thereby increases interpretability.

In contrast to deep supervised models, deep unsupervised models thus have the advantage that they are, to some extent, interpretable. They learn a latent representation that can be visually inspected.

While the reduced dimensionality of learned latent representations allows for, e.g., better studying how a genotype or haplotype affects the cellular state, i.e., helps interpret the data, this comes at the price of opaqueness. Thus, we partially lose the relationships between genes or genomic loci and the learned latent representation since latent representations are non-linearly associated with the observed variables, e.g., gene expression levels. However, this is important, e.g., in the development of therapeutics, where we need to know the genes that we want to target.

**Fig. 5:**
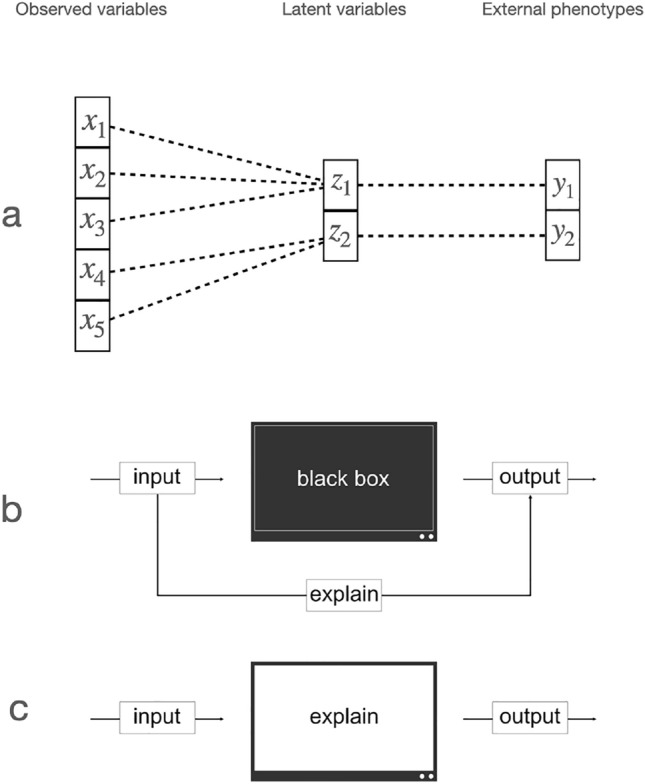
Taxonomy of interpretable deep learning models. **a** Learning relations between latent and observed variables as well as external phenotypes. Learning disentangled representations by model-based approaches enforces that individual latent variables are only related to a subset of observed variables or phenotypes. **b** A schematic illustration explaining post-hoc interpretability. The model is still a black box, i.e., the inner processes are not interpretable, but the influence of the observed variables on the output can be explained. **c** An illustration of model-based interpretability. Here, the model is adapted to the extent that the inner processes are interpretable. This is usually accompanied by a reduction in the performance of the model

In addition, although the estimated latent space is rather low dimensional, different latent variables in the latent space might be related to an external phenotype. Consequently, it would be desirable if variability in external phenotypes would be explained by mutually exclusive latent variables (see Fig. 5a).

Being able to infer the above-mentioned two relationships between latent variables and observed variables (genes) as well as between latent variables and external phenotypes increases the interpretability of DGMs.

A different yet frequently synonymously employed term is ”explainability”. Explainability is closely related to causality and consequently addresses, in the case of an unsupervised setting, the question of why samples are more or less similar to each other (Montavon et al. 2018). The ”why” in the case of omics data means that we are interested in obtaining the variables (e.g., genes) and also combinations over these, which are affecting the distance of data points. From this definition, we can see that interpretability is required for explainability.

### A taxonomy of approaches for interpretable deep learning models

Murdoch et al. (2019) present a general overview of interpretable AI approaches. They discriminate between model-based interpretability, where the model itself is designed to yield interpretable results, and post-hoc interpretability, where information is extracted from a potentially non-interpretable model (Fig. 5b,c). They also discriminate between global (dataset-level) and local (prediction-level) interpretation. While global interpretability relates to the features (variables) that are overall important, local interpretability relates to the determination of features that explain the predictions made for a given observational unit, e.g., a patient. In unsupervised models, the predictions are the coordinates of an observation in the latent space. Consequently, a local interpretation derived from a deep generative model should indicate how levels of observed variables respond to a change in the values of a latent variable for a specific observation.

As shown in the previous section, DGMs can estimate the parameters of the distribution of a low-dimensional representation of the original data. Having a distribution allows in addition to describing high dimensional observations to also assessing uncertainty of the observations in the latent space. This unique feature of generative approaches allows for estimating generalizability to different scenarios and for quantifying how well a specific observation is supported by the model.

With respect to interpretability, the same rules applicable to non-generative deep approaches also apply to DGMs. However, since DGMs learn a distribution over observed and latent variables, they offer additional opportunities due to their potential to generate new synthetic observations.

In the following, we give some examples for model-based and post-hoc approaches for interpreting the results from DGMs.

### Model-based approaches

Model-based interpretability refers to models that incorporate mechanisms that allow direct interpretation of learned relationships (Fig. 5c). However, the gain in interpretability is often accompanied by a loss in predictive accuracy or a lower support for the modeled data, i.e., lower likelihoods (Murdoch et al. 2019). The most prominent example of such an approach is a linear regression model, where one can directly interpret the models’ parameters. For increased capability to precisely model the data, additional terms, e.g. to model interactions between the variables, need to be incorporated into the model which subsequently impairs interpretability.

Taking the opposite path, complex deep learning approaches are modified to enforce, e.g., linearity or monotonicity (Molnar 2020).

Disentanglement strategies play a prominent role in making deep generative models more interpretable. Here, the goal is to learn latent representations that are, to a large degree, independent from each other. PCA, for example, is to a large extent frequently employed for deriving interpretable representations because the PCs are statistically independent from each other. This increases interpretability because it allows for annotation of the latent space, e.g., with external phenotypes. If multiple latent variables were linked with an external phenotype, the relationship would be fuzzy. Put differently, disentanglement strategies allow for learning an interpretable factor of variation (Fig. 5a).

Lotfollahi et al. (2021) propose the compositional perturbation auto-encoder (CPA), which learns a disentangled representation associated with, e.g., cells and genetic perturbations. The authors use an adversarial loss to separate latent cell representations from other covariates. More specifically, the model learns neural networks that try to discriminate between covariate information, such as genetic perturbations, from the learned latent representation. If the classification yields poor results, it is assumed that there is little or no information about the covariates in the learned representation; hence cell representations and representations associated with genetic perturbations would be disentangled and, therefore, easier to interpret. Another example of the use of disentanglement strategies is shown in Kinalis et al. (2019). Here the authors use a mixture of model-based and post-hoc interpretability by modifying an auto-encoder to include an orthogonality constraint in the loss function. This constraint ensures that the model learns disentangled representations, which can then be interpreted using saliency maps. More precisely, the authors visualize the influence of a small change in the expression of a gene on the representation. The authors also state that they can use this model to identify gene signatures of biological pathways.

Biological pathways are a prominent example of an underlying latent structure within noisy gene expression measurements. In Gut et al. (2021), the authors present pathway module VAE (pmVAE), which incorporates prior knowledge about biological pathways into the network architecture of a VAE and thus aids interpretability by learning latent representations that are factorized by pathway membership. This approach comes with the disadvantage that prior biological information is necessary, which might not always be the case. Another model, which relies on prior knowledge from pathway databases like Reactome (Jassal et al. 2020), that aids interpretability is presented in Rybakov et al. (2020). Here, the authors use VAEs with a regularized linear decoder to explain the variation of annotated factors from pathway databases, unannotated factors, and factors assumed to represent technical confounders.

DGMs also allow us to infer statistical relationships between the inferred latent variables and the observed variables. In an application to scRNA-seq data, Yu and Welch (2021) combine VAEs and GANs to learn disentangled representations, which showed, e.g., that a specific latent variable was related to immune function. Specifically, they show that a particular latent variable has learned the mesenchymal-epithelial transition using their model.

Unlike the previous approaches, Märtens and Yau (2020) propose to adapt the decoder network to decompose the variability for each gene into multiple components. More precisely, the model learns to separate the fraction of explained variance for each gene into variance explained by the latent variables, variance explained by additional covariates, and variance explained by their interaction effect. Here, the statistical relationship between latent and observed variables is given by a variance decomposition. Such a decomposition can be found, for example, in the classical analysis of variance (ANOVA). The authors demonstrate their method’s effectiveness in an application to bone marrow derived dendritic cells from multiple time points. Eventually, they decompose the temporal effects, effects due to a particular stimulus, and interactions between the stimulus and the time variable.

A substantial contribution to biology and medicine that disentangled, and thus interpretable, representations can make are predictions of changes in gene expression under previously unobserved drug treatments. One approach to do this is the so-called latent space vector arithmetics. In Yu and Welch (2021), e.g., the effects of drug treatments were predicted for previously untreated cell types by determining the average difference of latent representations for a given cell type under other drug treatments. This difference is then combined with the untreated cell type to predict changes in gene expression. A similar approach was used by Lotfollahi et al. (2019) to predict the perturbation response observed on mouse data for human cells. Similarly, Lotfollahi et al. (2021) model previously unobserved drug doses and drug responses, which biologists could use to optimize, e.g., experimental design. Although these methods involve strong assumptions, there is a large potential for biological and medical applications.

### Linearly decoded variational autoencoder (LDVAE)

An intuitive way to make deep learning methods interpretable is to look at the last layer of a neural network in the linear form (Lopez et al. 2020). Similarly, Svensson et al. (2020a) try to regain interpretability through a linear decoder in scVI (Fig. 3). Every cell represented by latent variables in the lower-dimensional latent space of scVI would typically be decoded to generate the corresponding parameters for each gene in each cell by using a negative binomial distribution. However, the low-dimensional latent representations are not easily interpretable. Hence, it is not possible to identify which genes influenced the representation of a particular cell. In many scenarios, e.g., eQTL studies, it is essential to know whether specific cell phenotypes are influenced by sets of genes that tend to be jointly expressed. This makes the interpretability of the latent space in the scVI framework essential. By integrating a linear factor model into the reconstruction part of the scVI framework–called linearly decoded variational autoencoder (LDVAE), Svensson et al. (2020a) show that they can use the flexibility of the non-linear representation of data without losing interpretability. However, in this model, a trade-off is made between the fit of the model and its interpretability, reflected in an increase in reconstruction error. The generative model of LDVAE remains very close to that of the classical scVI framework. However, one of the decoder networks, which estimates the expected number of transcripts per gene, is replaced by a linear factor model. Due to the linearity of the decoder, interpretability can be secured. This linear factor model assigns a weight to each gene in each cell, making the influence of this gene on the representation of the cell intelligible (Svensson et al. 2020a). Factor analysis, a generalization of the well-known principal components analysis, provides a generative model based on linear Gaussian latent variables (Murphy 2022). In factor analysis, the genes are modeled as linear combinations of the latent variables with an added error term. These linear combinations of several correlated genes are also referred to as meta-genes in the literature (Raychaudhuri et al. 1999; Brunet 2004; Svensson et al. 2020a). LDVAE provides model-based interpretability because it allows direct insight into the relationships between latent variables and input data through the linearity enforced on the decoder. Additionally, Svensson et al. (2020a) state that the proportion of variance explained by each latent variable can be computed, which brings additional advantages for interpretability. Being able to assign to each gene a factor loading allows performing an in-depth investigation of, e.g., pathway activity. We extracted the gene set corresponding to the B cell receptor signaling pathway, natural killer cell mediated cytotoxicity pathway, and primary immunodeficiency pathway from the KEGG database. Next, we took the maximum of the absolute factor loadings for each gene across all latent variables to compare for which pathways the LDVAE has learned an increased activity (See supplementary Notebook).

**Fig. 6:**
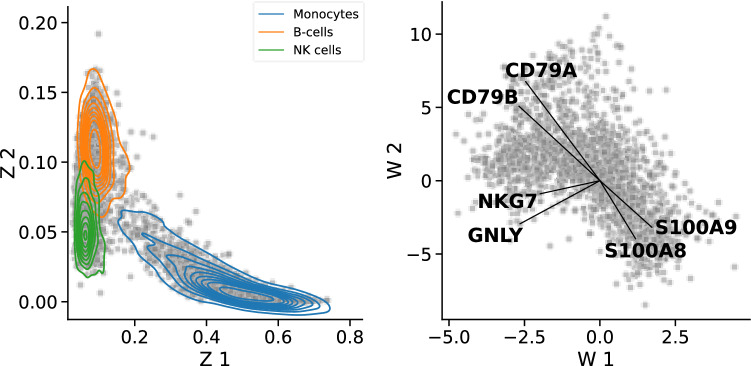
Interpreting latent representations using linearly decoded variational autoencoders (LDVAE). By using the LDVAE approach on the PBMC dataset (Zheng et al. 2017), we can visually examine the learned latent representations, here the first two variables. The color-coded kernel density estimates show the arrangement of three cell types (monocytes, B-cells, and natural killer (NK) cells) (left). The factor loadings of the first two variables can now also be displayed visually (right). Since the first two latent variables (Z 1 and Z 2) learned by the LDVAE approach separate the three cell types, they can be directly linked to factor loadings of co-expressed genes. Hence, variation along the Z 1 axis corresponds to variation in expression of CD79A and CD79B, whereas variation along the Z 2 axis corresponds to variation in expression of NKG7 and GNLY

To illustrate the utility of LDVAE, we show an example analysis of scRNA-seq data from peripheral blood mononuclear cells (PBMC) (Zheng et al. 2017). We can use LDVAE to infer gene programs from the data. More specifically, the model learns a latent representation of cells where cells with similar transcriptomes are grouped together and potentially depict gene expression phenotypes. However, due to the non-linearities in the networks of scVI, information about each gene’s influence on these latent representations stays hidden to the user. Using LDVAE, we can infer the relationship between gene weights and latent variables, which gives us a way to interpret the influence of one or several genes on the complex gene expression phenotypes (Svensson et al. 2020a). We train the network on the 2000 most highly variable genes and 7480 cells. The latent factors separate multiple cell types, all of which were defined by marker genes in Zheng et al. (2017) (Fig. 6). We can infer the corresponding information about co-expressed genes from the factor model’s loadings. More specifically, loadings for S100A8 and S100A9 indicate monocytes, loadings for CD79A and CD79B mark b-cells (Hu et al. 2020), GNLY and NKG7 represent a cluster of natural killer cells (Fig. 6). The model can infer gene programs by examining more latent dimensions for extracting co-expressed genes (Svensson et al. 2020a).

The learned lower-dimensional representations can be used as covariates in linear models to account for, e.g., confounding factors. In a recent preprint examining workflows to sc-eQTL mapping, the authors used LDVAE in a comparison with other methods to adjust their analyses for batch effects, and potentially unknown covariates (Cuomo et al. 2021). LDVAE did not perform particularly well in this setting. However, we believe that more elaborated interpretable deep learning approaches might be used to account for potential confounding factors in sc-eQTL studies in the future.

### Post-hoc approaches

In contrast to model-based interpretability, post-hoc approaches (Fig. 5b) have the advantage that they are applicable to any kind of model, irrespective of the detailed make-up. This also implies that post-hoc approaches, in contrast to model-based approaches, do not have to be tailored to the specific properties of omics data.

Post-hoc approaches infer feature importance by approximating the non-interpretable model with simpler models. The most popular post-hoc approaches employ an additive model to approximate the output (the predictions or the coordinates in a learned low-dimensional embedding) of the complex model. The output of the interpretable model then is the effect of the individual input variables on the output of the complex model.

The input variables are usually binarized where 0 represents an absence, or a reference value, while 1 the original value of the variable. The effect of the input variables on the complex models’ output is then assessed by perturbing the input data.

Popular approaches are Local Interpretable Model-agnostic Explanations (LIME) (Ribeiro et al. 2016), the SHapley Additive exPlanation (SHAP) (Lundberg et al. 2018) approach, e.g., demonstrated in Lundberg et al. (2020) and visualization techniques such as Layer-wise Relevance Propagation (LRP) (Bach et al. 2015; Montavon et al. 2017).

LIME provides a local explanation based on samples of similar observations around a given observation whose prediction is to be explained. The result is a locally interpretable model in which we can assess the impact of the input variables on the prediction or latent space information we want to explain.

LRP is specifically tailored to deep neural networks and can identify the observed variables which are activated in an observation, relative to a reference state, and which respond sensitive to a change in the prediction made by the NN. LRP has been employed, e.g., in Chereda (2021) for prediction of metastasis in breast cancer.

In the SHAP approach, marginal feature importance on predictions is inferred by removing different variables when fitting the simple additive model. This implies that a multitude of different combinations of input variables have to be investigated. Due to the resulting combinatorial complexity, SHAP is computationally intensive but has been frequently applied for interpretable deep learning with omics data, e.g., for inferring gene importance on predictions of survival (Kuruc et al. 2021) and clustering in combinations with AEs (Lemsara et al. 2020).

While the aforementioned approaches have been successfully applied to supervised networks, they might generally be applied to DGMs. In fact, DGMs can easily generate new, perturbed observations, which is a building block of many post-hoc approaches. Moreover, since DGMs represent a model for the joint distribution of latent and observed variables, the perturbation process can be controlled very well. Recently, VAEs have been reported to improve the interpretability of the LIME approach (Schockaert et al. 2020).

### Extracting patterns with log-linear models

**Fig. 7:**
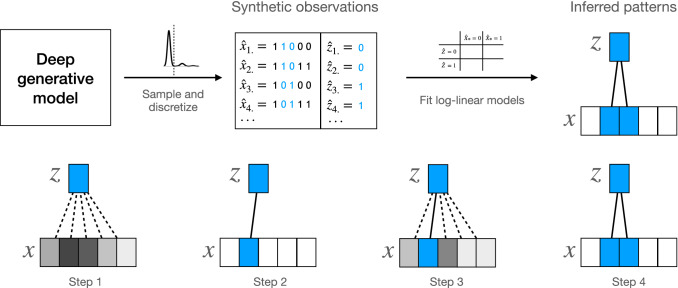
Extracting joint patterns between observed and latent variables with log-linear models. A deep generative model such as a variational autoencoder (VAE) as employed by scVI is trained on single-cell RNA-Seq data. After training, synthetic observations for observed and latent variables (${\hat{x}}$ and ${\hat{z}}$, shown are four observations) are sampled from the posterior or the prior distribution of the latent variables *z*. Synthetic observations are further discretized, which is straightforward in the case of single-cell RNA-Seq data where usually bimodal distributions are observable. Log-linear models are step-wise fit to the discretized synthetic observations to identify joint patterns between latent variables and observed variables (the genes). Starting with no selected observed variables, the association of the latent variable with all observed variables is inferred (strength of association indicated by grey color; step 1). The variable with the strongest association is then added to the model (step 2). In the following iterations, steps 3 and 4 are repeated until a given number of variables has been selected. In this example, we stop after step 4 since all information-carrying observed variables (2 and 3) have been selected

One post-hoc approach which has been recently proposed by us (Hess et al. 2020) is able, to infer the relationship between observed variables and latent variables. Being a post-hoc approach, it is applicable to any kind of DGM such as DBMs, VAEs and GANs. Specifically, the approach only requires synthetic observations for the observed and latent variables sampled from a trained DGM. In contrast to LDVAE, where relations between latent and observed variables are derived by simplifying the decoder, our post-hoc approach works by simplifying the distribution of observed and latent variables. Specifically, observed and latent variables are discretized, and the co-occurrence of the derived discrete states for observed and latent variables are investigated in high dimensional cross tables (Fig. 7). For the analysis of cross tables, log-linear models are employed, which are related to Poisson regression. Specifically, a linear model is employed to study potentially higher-order interactions between latent and observed variables. By fitting log-linear models step-wise, an observed variable is added if the interaction with already included observed variables conditional on the state of latent variables is higher than all others until to that point not selected variables. By considering the interactions between observed and latent variables, we are able to infer patterns over observed variables that contribute to the essential structure in the data. This renders the approach specifically interesting since it allows for prediction-level interpretability. This means that different observations, e.g., different cells, can be compared in terms of their similarity in the latent space, and the patterns in the observed variables can be subsequently studied to infer the essential differences between the cells (see also the supplementary Jupyter notebook for an exemplary analysis).

**Fig. 8:**
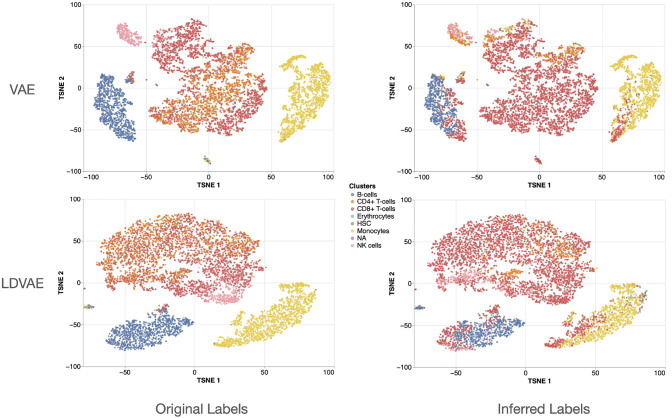
Annotating synthetic observations derived by scVI based on patterns in extracted genes. Shown are samples from the posterior inferred by scVI from the PBMC data in a two-dimensional embedding derived by t-stochastic neighbor embedding (t-SNE) (Van der Maaten and Hinton 2008). VAE and LDVAE are employed. Based on the samples from the posterior of VAE and LDVAE, the log-linear approach (Fig. 7) was employed to extract eight genes that form joint patterns with the latent variables. Samples drawn from the posterior distribution were then assigned to a real expression vector and the corresponding cell type label, based on the pattern in the eight extracted variables (Inferred Labels). For comparison, samples from the posterior are also shown colored by the original cell type label (Original Labels). The extracted genes were FCN1, IGLC3, SH2D1B, GZMK, MEG3, KLRB1, NELL2, S100A12 for VAE and S100A8, IGKC, LYAR, FCGR3A, GPR183, COTL1, ARHGAP44, AQP3 for LDVAE

In an exemplary application, we infer a latent representation of single-cell RNA-Seq data using scVI (Lopez et al. 2018) (see also section ”Single Cell Variational Inference (scVI)”). We employ the same data-set as used for the application of LDVAE. Using the approach described in Hess et al. (2020), we then extract eight genes which form joint patterns with latent variables. To demonstrate that the extracted variables contribute to the essential structure in the data, we annotate samples from the posterior distribution of the VAE based on patterns identified in the eight variables. Specifically, we transfer a label of an observed cell-type to the sample, by pattern matching in the eight identified variables. As also demonstrated in Hess et al. (2020) these genes carry enough information to annotate the samples generated from scVI (Fig. 8). We observe some deviations compared to using the original labels, but overall the synthetic cells are correctly annotated. We employ both the original VAE as described by Lopez et al. (2018) as well as the LDVAE, which lacks hidden layers in the generator network (Fig. 3). We observe that the samples generated by the LDVAE allow for a slightly worse separation of cells compared to the VAE. The identified genes which carry essential information do now allow us, e.g., to compare the individual cells in terms of their expression pattern in the identified genes. In the supplementary Jupyter notebook you can find a comparison of cell-types in terms of their expression profile in the identified genes.

## Conclusion

DGMs are a versatile approach for extracting latent representations from omics data. As they are unsupervised approaches, they learn the joint distribution of the data, which allows for addressing uncertainty and better generalizability. This permits an improved integration of different population samples as well as data modalities or imputation of missing observations. A further promising application is the generation of counterfactual observations, which are important in assessing causality in observational studies as was done in, e.g., Louizos et al. (2017) and Parbhoo et al. (2018). With respect to explainability, e.g., to infer why two cells are more similar/dissimilar, DGMs thus are promising tools.

We described different approaches of rendering DGMs more interpretable, which extend the models beyond a better description of the relationship between data points in terms of their overall similarity. Some approaches target interpretability by enforcing disentangled latent representations so that each latent variable is interpretable with respect to a given external phenotype. Another group of approaches is concerned with inferring the relationship between observed and latent variables.

In the following, we discuss the applicability and indicate challenges that are still to be resolved.

### Model-based vs. post-hoc approaches for interpretability

The advantage of post-hoc approaches over model-based ones is their larger flexibility. It is possible to employ the best performing approach and only in the second step care about the interpretations (Samek et al. 2021). On the other hand, the risk in post-hoc approaches is that the model, due to uncontrollable noise, focuses on artifacts that hinder learning useful information and naturally also the subsequent interpretation (Laugel et al. 2019).

Model-based interpretability approaches have the disadvantage that they are, by definition, adapted to a specific model class and thus cannot be used flexibly. Additionally, model-based interpretability often leads to reduced predictive performance or increased reconstruction errors as described by Murdoch et al. (2019) and Svensson et al. (2020b). Additionally, see also our results presented in Fig. 8 where the samples generated by LDVAEs, which are adapted for model-based interpretability, do less accurately reflect the different cell types compared to unmodified VAEs.

### Advantages/disadvantages of different generative approaches

To date, most approaches for interpretable generative modeling are focused on VAEs. The variational Bayes approach of VAEs allows for robust and time-efficient estimation of parameters. In contrast, DBMs are less frequently used because of the time consuming Gibbs sampling procedure. However, the DBM is an otherwise versatile approach because it allows for flexible sampling schemes. Compared to VAEs, there are also fewer approaches which employ GANs to extract interpretable representations. This is mainly because drawing latent space information conditional on information for the observed variables (the posterior distribution in VAEs) is not straightforward to conduct (see GAN architecture in Fig. 3). However, GANs can generally create more accurate samples compared to VAEs (Goodfellow et al. 2016). Therefore, Yu and Welch (2021) have proposed combinations of VAEs and GANs. They employ VAEs to generate disentangled latent representations, which are subsequently employed in the generator of the GAN to generate synthetic samples of high quality.

### Challenges

#### Training data and hyperparameter optimization

Approaches like scVI can generally yield an improvement over shallow approaches such as PCA (Raimundo et al. 2020). Although there is not always a benefit in using deep models over shallow approaches Bellot et al. (2018), in theory, deep approaches should always result in a better performance given enough data being available. However, compared to simpler approaches like PCA, DGMs require tuning hyper-parameters, e.g., the architecture of the network or the learning rate. Still, there is no established way to do so.

#### Suitable error model

Although interpretable AI methods hold great promise for automated analysis of complex biological data, it is still necessary to adapt the corresponding models to the given data structures. This also means that the technical noise introduced during experiments must be understood, which continues to require subject-matter expertise. Also, one should note that some of the deep generative models have been shown to have difficulties in learning bimodal distributions, which are common in scRNA-seq data (Treppner et al. 2021). Additionally, it has been demonstrated by Breda et al. (2021) that scVI often falsely identifies genes as co-expressed. This could also affect LDVAE and result in incorrect gene programs being identified.

#### Inferring significance of statistical associations between observed and latent variables

Although a number of the investigated approaches allow for extracting relations between learned latent and observed variables as described e.g., in Märtens and Yau (2020), there is still a large amount of research to be performed in detecting significant associations. For instance, in both the LDVAE approach by Svensson et al. (2020b), and the log-linear modeling approach by Hess et al. (2020) it remains challenging to infer a cutoff defining meaningful association of observed and latent variables. Hess et al. (2020) proposed to employ permutation-based approaches. Even though they are effective, they are also very time-consuming and only feasible for a small number of variables. The main problem in applying statistical testing here is the inevitable dependency between observed and latent variables for which no closed-form or time-efficiently evaluable density function is available.

#### Framework for evaluation and validation of extracted information

What is generally missing is a framework for rigorous evaluation of the explanatory performance. As already pointed out by Murdoch et al. (2019), there are currently very few approaches for evaluating methods for interpretable machine learning. Hence, it is still challenging to compare different approaches and judge the scale of their impact. Also, it is difficult for researchers to choose an appropriate method without proper evaluation methods.

As a solution, it is proposed to generate data based on the knowledge extracted from interpretable generative approaches, which could then be employed to test the respective models. Another approach suggested by Murdoch et al. (2019) could be to use the extracted information for solving real world problems. For example, consider the log-linear approach described by Hess et al. (2020). The proposed log-linear models allow to extract the statistical associations between observed (genes) and latent variables. Consequently, we receive a model describing the similarity of cells with a small number of genes. This model could easily be evaluated on other data sets in order to check if cell-type similarities are correctly inferred.

To improve model-based approaches, Murdoch et al. (2019) further suggest to use exploratory data analysis. This could involve exploring relationships learned from the models, for example, through visualizations. For instance, in Kinalis et al. (2019), saliency maps, a method for visualizing prominent features, are used to visualize the influence of Hallmark molecular pathways on latent representations. Murdoch et al. (2019) also suggest that the user could correct false correlations learned by interpretable methods. In a biological context, for example, it is conceivable that latent variables representing a particular pathway could be compared with prior knowledge from databases to generate feedback for the model.

In biology, more specifically, the analysis of omics data, there is the possibility of experimental validation of the putative findings from interpretable deep generative models. As this research area is still at an early stage, there are very few studies in which rigorous experimental validation is performed. However, both the methods and the potential users could benefit from this by adjusting model predictions to experimental results and giving users a better overview of the utility of the respective techniques in their application areas.

## Data Availability

The accompanying data can be found at https://github.com/ssehztirom/interpretable-generative-deep-learning.

## Code availibility

The accompanying Jupyter notebooks can be found at https://github.com/ssehztirom/interpretable-generative-deep-learning.
